# Monthly intravenous methylprednisolone in relapsing-remitting multiple sclerosis - reduction of enhancing lesions, T2 lesion volume and plasma prolactin concentrations

**DOI:** 10.1186/1471-2377-6-19

**Published:** 2006-05-23

**Authors:** Florian Then Bergh, Tania Kümpfel, Erina Schumann, Ulrike Held, Michaela Schwan, Mirjana Blazevic, Axel Wismüller, Florian Holsboer, Alexander Yassouridis, Manfred Uhr, Frank Weber, Martin Daumer, Claudia Trenkwalder, Dorothee P Auer

**Affiliations:** 1 Section of Neurology, Max-Planck-Institut für Psychiatrie, München, Germany; 2 Section of Neuroradiology, Max-Planck-Institut für Psychiatrie, München, Germany; 3 Sylvia Lawry Center for Multiple Sclerosis Research, München, Germany; 4 Department of Diagnostic Radiology, Ludwig-Maximilians-Universität, München, Germany; 5 Section of Neuroendocrinology, Max-Planck-Institut für Psychiatrie, München, Germany; 6 Section of Satistics, Max-Planck-Institut für Psychiatrie, München, Germany; 7 Section of Clinical Chemistry, Max-Planck-Institut für Psychiatrie, München, Germany; 8 Klinik und Poliklinik für Neurologie, Universität Leipzig, Leipzig, Germany; 9 Institute of Clinical Neuroimmunology, Klinikum Großhadern, Ludwig-Maximilians-Universität, München, Germany; 10 Paracelsus-Klinik, Kassel, Germany; 11 Department of Neuroradiology, University of Nottingham, UK

## Abstract

**Background:**

Intravenous methylprednisolone (IV-MP) is an established treatment for multiple sclerosis (MS) relapses, accompanied by rapid, though transient reduction of gadolinium enhancing (Gd+) lesions on brain MRI. Intermittent IV-MP, alone or with immunomodulators, has been suggested but insufficiently studied as a strategy to prevent relapses.

**Methods:**

In an open, single-cross-over study, nine patients with relapsing-remitting MS (RR-MS) underwent cranial Gd-MRI once monthly for twelve months. From month six on, they received a single i.v.-infusion of 500 mg methylprednisolone (and oral tapering for three days) after the MRI. Primary outcome measure was the mean number of Gd+ lesions during treatment vs. baseline periods; T2 lesion volume and monthly plasma concentrations of cortisol, ACTH and prolactin were secondary outcome measures. Safety was assessed clinically, by routine laboratory and bone mineral density measurements. Soluble immune parameters (sTNF-RI, sTNF-RII, IL1-ra and sVCAM-1) and neuroendocrine tests (ACTH test, combined dexamethasone/CRH test) were additionally analyzed.

**Results:**

Comparing treatment to baseline periods, the number of Gd+ lesions/scan was reduced in eight of the nine patients, by a median of 43.8% (p = 0.013, Wilcoxon). In comparison, a pooled dataset of 83 untreated RR-MS patients from several studies, selected by the same clinical and MRI criteria, showed a non-significant decrease by a median of 14% (p = 0.32). T2 lesion volume decreased by 21% during treatment (p = 0.001). Monthly plasma prolactin showed a parallel decline (p = 0.027), with significant cross-correlation with the number of Gd+ lesions. Other hormones and immune system variables were unchanged, as were ACTH test and dexamethasone-CRH test. Treatment was well tolerated; routine laboratory and bone mineral density were unchanged.

**Conclusion:**

Monthly IV-MP reduces inflammatory activity and T2 lesion volume in RR-MS.

## Background

Intravenous methylprednisolone (IV-MP), 500–1000 mg for 3–5 days, is an established treatment for acute relapses of multiple sclerosis, speeding recovery [1,2]. It does not prevent future relapses. However, a study in isolated optic neuritis suggested that a single course of IV-MP may delay the occurrence of another episode of inflammatory demyelination and thus conversion to definite MS according to Poser's criteria [3,4]. Repeated administration of IV-MP in regular intervals may extend these beneficial effects, but this approach has not been fully evaluated and requires further study [5]. In secondary progressive MS, IV-MP every two months showed partial, dose-dependant benefit in several secondary endpoints [6]. In relapsing-remitting MS, a five-day course of IV-MP every four to six months slowed progression of MRI measures of brain atrophy, as compared to treatment for relapses only [7].

The mechanisms underlying the beneficial effects of corticosteroids in MS remain incompletely understood. Rapid reduction of inflammatory blood-brain-barrier (BBB) disruption, as shown by disappearance of gadolinium (Gd) enhancement after few days of treatment [8-10], is a prominent effect. Corticosteroids are also potent immunomodulatory drugs, inhibiting the expression of inflammatory mediators (including TNF and leukocyte-endothelial cell adhesion molecules) and cellular immunity (reviewed in [11]). Further, the endocrine and immune systems closely interact (reviewed in [12]), with ACTH, cortisol, prolactin (PRL) and growth hormone (hGH) being most often implicated in the regulation of the immune system. We [13,14] and others [15] have described a disturbed regulation of the hypothalamo-pituitary-adrenal (HPA) axis in MS, as well as hyperprolactinemia during acute attacks [16]. While causal relations are at present difficult to establish, corticosteroids do affect the secretion of several other hormones, and may thus have indirect effects on the immune system.

It is unknown if these effects remain active with regular administration. In order to study the effect of pulsed corticosteroids on several of these aspects, we administered a single infusion of MP, once per month, to RR-MS patients. Based on guidelines derived from natural history and treatment trial data [17,18], we designed a single-cross-over study with a six-month baseline and a six-month treatment period, and determined the impact on

1. inflammatory disease activity as shown by BBB disruption (frequency of Gd-enhancing lesions on brain MRI),

2. total T2 lesion load,

3. plasma hormone concentrations and dynamic measures of neuroendocrine regulation, and

4. selected plasma markers of immune function.

## Methods

### Study design

Participation in this open-label, single-cross-over study was offered to patients with clinically definite [19], relapsing-remitting MS, followed in a natural history study with monthly Gd-enhanced MRI. Patients could enter that study no earlier than three months after the last relapse or course of IV-MP. In each individual patient, scans were performed at the same time of the day, and blood was drawn from an antecubital vein before each scan. We first selected those patients who had at least one Gd-enhancing lesion on the first three scans, and continued monthly visits for another three months. Final inclusion in the treatment trial required that the first six monthly scans showed at least three Gd-enhancing lesions (i.e. a mean of 0.5 Gd+ lesions per scan). Further inclusion criteria were: one or more relapses during the two years before the first MRI scan, contraindication to immunosuppressive or immunomodulatory therapy or decline of these therapies by the patient, and no contraindications to glucocorticoid treatment. Upon inclusion, monthly MRI and blood sampling was continued until month 12. Treatment started after the MRI at month 6 (see below).

The study was performed and funded by the participating institutions, with no external funding. It was approved by the ethics committee of the Bavarian State Board of Physicians (Bayerische Landesärztekammer). Patients gave written informed consent.

### Patients

**Figure 1:**
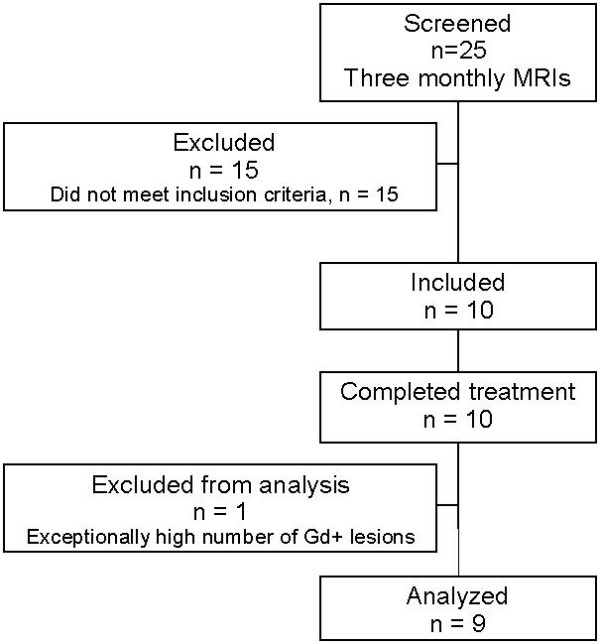
Study flow chart

Twenty-five patients were screened by three monthly MRI scans. Ten patients fulfilled the inclusion criteria, but we excluded one patient with a very high baseline activity to reduce the risk of regression to the mean. See table 1 for clinical and demographic characteristics of the finally included nine patients, and figure 1 for a study flow chart. None of the patients had ever been treated with immunosuppressive or immunomodulatory drugs; mean annualized relapse rate during the entire disease duration was 0.68, and during the two years prior to study inclusion, 0.8.

### Magnetic resonance imaging

Once every month, brain MRI was performed on the same 1.5T Signa Echospeed (GE Medical Systems), including axial T1-weighted sequences (TR = 640 ms, TE = 14 ms, 4 mm thick, 1 mm gap) as well as a proton density and T2 weighted dual fast spin echo sequence (TR = 3600 ms, TE = 13 and 91 ms, resp.) and a FLAIR sequence (TR = 10 s, TE = 133 ms, TI = 2200 ms) inherently coregistered with the T1 axial scans. Axial and coronal (3 mm thick, 1.5 mm gap) T1-weighted sequences were acquired after i.v. injection of 0.1 mmol/kg gadolinium chelate (Omniscan(R)), with the interval between injection and first axial scan standardized at 6 minutes.

The number of Gd-enhancing lesions on brain MRI was determined by a blinded radiologist (E.S.) on the scans presented in random order, with the date of acquisition masked. For each patient, the mean number of active lesions per scan was calculated for baseline and treatment period. To determine lesion volume and BFV, a neural network based image analysis system was used as described [20]. Briefly, data preprocessing included manual delineation of the intracranial cavity contents, automatic grey value correction (coil inhomogeneity and interslice differences) and extraction of a small training data subset by interactive contour tracing of regions representing the structure classes: normal appearing grey matter (GM), normal appearing white matter (WM), cerebro-spinal fluid (CSF), and MS lesion. A hierarchical vector quantization approach was then deployed to segment the multi-dimensional image data into five classes: GM, WM, CSF, lesions and other (comprising meninges, vessels). The BFV was determined as the ratio of pixels belonging to either brain, i.e. GM, WM or MS lesions, to those belonging to brain or CSF. CSF segmentation was based on the image information obtained by all acquired MRI sequences; MS-lesions were identified based on T2, PD, and FLAIR data. The segmentation performance compared favorably (higher retest stability) with the more standard region growing segmentation algorithm for T2 lesion load (T2-LL) and angle image [21] for BFV [20]. T2-LL and BFV were calculated for four time points (start and end of baseline: months 1 and 6; and four weeks after the first and the last MP infusion: months 7 and 12).

### Treatment

From month six to eleven, patients received 500 mg MP i.v. after each MRI scan, followed by an oral tapering dose of 40, 20 and 10 mg MP for one day each to prevent any potential temporary glucocorticoid deficiency.

### Serum cytokine and hormone concentrations

Blood was collected, between 15:00 and 18:00 in the afternoon and always before the MRI scan, into tubes containing EDTA and trasylol, and plasma was stored at -80 degC until processing. sTNF-RI, sTNF-RII, IL-1ra and sVCAM1 were quantified by commercial enzyme-linked immunosorbent assay (ELISA) kits (s-TNF-RI and RII: Biosource Europe, Belgium; IL-1ra and VCAM-1: R&D Systems, Minneapolis, USA). Hormone concentrations were measured using commercial radioimmuno (RIA) or chemiluminescence assays (cortisol: RIA, DRG Instruments, Germany; ACTH: RIA, and prolactin: Chemiluminescence Immunoassay, both Nichols Institute Diagnostics, San Juan Capistrano, CA, USA).

### Dexamethasone-CRH test

The dexamethasone-CRH test (Dex-CRH test) was performed, and values were derived, as described [13], once before the start of treatment, and once between months 11 and 12.

### Safety

In addition to a full physical and neurological examination including EDSS [22], blood chemistry (including calcium, phosphate and vitamine D3), blood count, thyroid function tests, urinanalysis and pyrrolidine cross-links in urine were determined monthly. At baseline and after six months of treatment, patients underwent the short ACTH test (i.v. injection of 200 microgram tetracosactid [Synacthen(R), Novartis] at 08:00, with determination of plasma cortisol concentration before and 60 minutes after injection) and CT densitometry of the lumbar spine. All procedures were performed on an outpatient schedule.

### Outcome measures

The mean number of Gd enhancing lesions per monthly MRI scan was defined as the primary outcome measure. In accordance with published guidelines and sample size estimates [17,18], we planned to enroll ten patients. Secondary outcome variables were T2 lesion volume and monthly plasma hormone concentrations. Serum immune variables and results of neuroendocrine tests were analysed in an exploratory fashion. Number of relapses, EDSS, routine laboratory results and bone mineral density measures were safety variables, with study termination criteria predefined for the laboratory results. Brain fractional volume (BFV) was determined to control for global changes in brain volume, potentially resulting in false results of T2 volume analyses; BFV was not an outcome measure.

### Data analysis

According to the data structure, the effect of treatment on the number of active lesions, on hormone and cytokine receptor concentrations was analyzed using the non-parametric Wilcoxon matched-pairs signed-ranks test and a one-factorial univariate analyses of variance (ANOVA) with repeated measures design. The continuous dependent variables were transformed with the ln-transformation [Y = ln (x+1)] before ANOVAs. Associations over time between the number of active lesions to plasma hormone concentrations and immunological parameters were analysed by cross-correlation analysis. Changes of T2-LL and BFV, and all other laboratory results were analyzed by MANOVA, and followed by univariate F-tests where appropriate. As nominal level of significance, p = 0.05 was accepted; it was corrected (according to Bonferroni correction procedure) for all post-hoc tests, in order to keep the type I error less or equal to 0.05. Data are given as mean +/- standard error of the mean (SEM).

### Comparison group

The comparison group was extracted from the database maintained at the Sylvia Lawry Center for Multiple Sclerosis Research. This database includes clinical and imaging information on patients examined according to clinical trial protocols at separate centers, including untreated control groups from several large clinical trials and natural history studies. Patients were selected based on the following criteria: A diagnosis of relapsing-remitting multiple sclerosis, no current immunosuppressive or immunomodulatory treatment, longitudinal Gd-MRI follow-up over twelve months, presence of at least 1 new Gd+ lesion during the first three months, and at least three new Gd+ lesions during the first six months. This yielded a group of 83 patients (55 females, mean age 34.4 +/- 0.8 years, mean duration of disease of 7.5 +/- 0.6 years). The MRI protocols applied 0.1 mmol/kg of gadolinium chelate. The mean interval between injection and post-contrast T1w imaging was 5.7 minutes; the delay was 5 minutes in 69 of the selected 83 patients, one minute in one patient, and between 7.5 and 15 minutes in the remaining patients. Monthly scans over the entire twelve-month period were available for seven patients; among the remaining patients, four to six scans over the first six-month-period were available for 75 patients, and for both six-month-periods for 57 patients. The mean number of new Gd+ lesions was calculated for each individual patient for the first and the second six-month-period and expressed as mean number of Gd+ lesions per scan.

## Results

**Figure 2:**
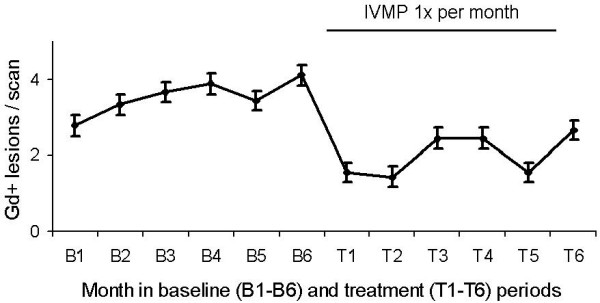
Time course of the mean number of active lesions on monthly Gd-enhanced brain MRI.

During the treatment period, the mean number of active lesions per scan was reduced in eight of the nine patients, by 14 to 100% compared to the baseline period; the number increased by 21% in one patient (table 1). The median rate of reduction was 43.8%, and the mean rate of reduction was 46.4%. The mean number of active lesions averaged over the group was reduced from 3.55 per month (baseline period) to 2.15 (treatment period, a reduction by 39.4%), statistically significant by Wilcoxon matched-pairs signed-ranks test (two-tailed p = 0.013). Analysis of variance also revealed a significant effect of time [Wilk's aver. multivariate test of significance; effect of time: F(11,88) = 2.15, p = 0.025]. The anti-inflammatory effect was apparent from the first scan on treatment (T1 in figure 2). Tests with polynomial contrasts in ANOVA revealed that the mean number of enhancing lesions can be optimally fitted by a polynomial curve of third order (effect of polynomial contrast of 3rd order: F(1,8) = 6.35, sig of F = 0.036).

The comparison group had a mean of 2.95 Gd+ lesions per scan during the first six months of observation. Over the next six months, the frequency of new Gd+ lesions per scan increased or decreased over a wide range in indidvidual patients, yielding a median rate of change of -14.3% (reduction), but a mean rate of change of +15.2% (increase). When analyzing the group averages, the mean number of new Gd+ lesions was reduced to 2.76 Gd+ lesions per scan (a reduction by 6.4%). These changes were not significant (Wilcoxon, p = 0.32).

In the study population, there was also a significant time effect on T2 lesion load (Wilk's multivariate test for effect of time, p = 0.002; table 2). Over the five-month baseline period, T2-LL increased by 13% (p < 0.001). The first MP infusion led to a decrease in T2-LL (by 8%, p = 0.021 for comparison of month 7 to month 6, the last baseline scan), and repeated MP infusions further decreased T2-LL until month 12 (p = 0.001 for comparison to month 7 or to month 6), representing a total decrease of 21% during the treatment period. The changes in BFV were not significant (p = 0.086; table 2).

IV-MP treatment exhibited a significant effect on mean plasma prolactin concentrations during baseline vs. treatment period, with lower concentrations during the treatment period (Table 3; Wilks multivariate test in MANOVA (F(1,8) = 7.32, p = 0.027). Cross correlation coefficients revealed significant associations of lag -1 between plasma prolactin concentrations and the number of active lesions on MRI (Cross correlation coefficient: r = 0.728, p < 0.05). This means that prolactin levels increase or decrease one month before a corresponding change in the number of active lesions is observed on MRI. Cortisol and ACTH did not differ significantly between the baseline and treatment period (table 3). Also, results of the dexamethasone-CRH test were unchanged (table 3). There was no significant effect of treatment on soluble TNF receptors, IL-1 receptor antagonist or sVCAM1 (which increased slightly; table 4).

Four relapses occurred during the baseline period (three treated by three-day courses of IV-MP, and MRI performed no earlier than ten days afterwards), and two mild relapses during the treatment period (no additional steroids administered). The relapses occurred in individual patients, and all recovered to the previous level of impairment. The mean EDSS score was almost unchanged between baseline (1.45 +/- 0.30) and end of study (1.40 +/- 0.37). Apart from occasional sleep disturbance on the day of infusion and mild flush-like sensations immediately following the infusion, treatment was well tolerated. Results of routine laboratory investigations were unchanged (not shown). All patients had an adequate rise of plasma cortisol after i.v. tetracosactid ("short ACTH test"), both at baseline and after six months of treatment (table 3). Biochemical measures of bone metabolism (calcium, phosphate, vitamin D3 in serum, and pyrrolidine cross-links in urine, data not shown) as well as bone mineral density (CT densitometry of the lumbar spine: 140.34 +/- 15.79 before, 135.50 +/- 15.93 after treatment) were not significantly changed.

## Discussion

We show here that a single monthly infusion of 500 mg methylprednisolone with a three-day oral taper can reduce inflammatory disease activity in patients with relapsing-remitting MS, without clinically relevant side effects. Our data support and extend two previous studies of pulsed steroid treatment in MS. In RR-MS patients, regular courses of IV-MP every four to six months, as compared to treatment for clinical relapses only, delayed accumulation of T1 hypointensities and brain atrophy [7]. Only a non-significant effect was seen on T2 lesion load, and the frequency of gadolinium-enhancing lesions was not assessed. In secondary analyses, disease progression appeared to be delayed, while there was no effect on the relapse rate. In secondary-progressive MS, repeated courses of IV-MP every eight weeks delayed the time to confirmed disease progression in a dose-dependant fashion, while failing with respect to the proportion of patients with disease progression (the primary outcome measure) [6]. MRI was not performed in that trial. IV-MP has previously been shown to rapidly, though transiently reduce gadolinium enhancement in MS, when given for several days for acute relapses [8-10,23]. Our study suggests that in the absence of relapse, a single infusion has a similar effect, and that the reduction of inflammatory activity can be extended by repeat infusion.

With the single cross-over study protocol and the selection of patients based on a minimum number of enhancing lesions during the baseline period, a potential alternative explanation for the reduction of enhancing lesions would be "regression to the mean". The mean number of active lesions during the baseline period is comparable to some earlier studies using the same single cross-over design as in this study, while it was lower in others [24-27]. Comparison to a pooled dataset showed that inflammatory activity in our patients was high, but not exceptional (see below). In addition, the sharpest decline in the slope of the regression curve occurs between months 6 and 7, concomitant with the start of treatment. These factors make pure "regression to the mean" highly unlikely.

In an effort to estimate the magnitude of regression to the mean, we extracted a comparison group from a large database of patients collected from various centers, who had undergone regular contrast enhanced MRI over twelve months as placebo patients of clinical trials. This comparison group of 83 patients, selected by clinical and imaging criteria equivalent to ours, had a mean frequency of Gd-enhancing lesions which is higher than would be expected for an unselected population of MS patients [28]. Of note, this number tends to even underestimate the true frequency of new lesions, since not all patients had strictly monthly scans, and some new lesions with a short duration of enhancement may have been missed. By natural history alone, the mean number of new enhancing lesions in the comparison group changed over a wide range during the following six months, resulting in a non-significant reduction by 6.4% for the entire group. Thus, many patients selected for a minimum frequency of active lesions appear to continue on that above-average inflammatory activity, and at least over a subsequent six-month period, "regression" to mean values as described in unselected MS patient cohorts [28] applies to the selected group only on a relatively limited scale. Importantly, even if we conservatively assume a more pronounced effect of regression to the mean in our patients, we can still conclude that monthly methylprednisolone contributed far more to the reduction of inflammatory activity.

### Corticosteroids and T2 lesion load

This study is the first to show a favorable effect of methylprednisolone on T2 lesion volume. A potentially confounding effect of the dehydrating properties of high-dose glucocorticoids, leading to pseudoatrophy [29,30], can be largely excluded because (i) no accompanying brain volume decrease was seen and (ii) serial MP infusions led to a cumulative decrease of T2 lesion volume. Previous studies over short [23,30,31] and longer periods [7] failed to demonstrate changes in T2 lesion load. In short-term studies of MP in acute exacerbations [23,30,31], an anti-edematous effect can be expected to be particularly prominent, while our patients received a single infusion in the absence of acute relapse. Edema resolution thus appears to contribute less to this change in T2 signal than other histological changes (extent of gliosis, macrophage infiltration or remyelination).

### The role of prolactin during corticosteroid treatment

The parallel time-course of prolactin and Gd-enhancing lesions during both baseline and treatment with repeated steroid infusions is intriguing with respect to a possible role of prolactin in the autoimmune process. Elevated plasma levels of prolactin have been described in MS relapse, with return to normal concentrations upon recovery [16]. In other studies, plasma levels were higher in MS patients than in controls, albeit in the normal range [32], or normal [33,34]. While the role of prolactin in human immune function is a matter of debate (e.g., [35,36]), experimental data appear to support an immunostimulatory effect of prolactin [37-39]. Elevated levels of endogenous or therapeutic glucocorticoids are associated with clinically relevant immunosuppression [40] through several mechanisms, and at the same time reduce expression and secretion of prolactin [41,42]. A mere side-effect of steroid administration is unlikely given the significant cross-correlation with enhancing lesions during the baseline period. It remains to be determined if lower prolactin during treatment contributes to the reduction of inflammatory disease activity, or if blunted inflammation leads to a reduced level of general stressors which non-specifically stimulate prolactin secretion.

### Measures of immune function

The panel of immunological analyses performed in this study was chosen based on specific hypotheses rather than being designed as a comprehensive immunological assessment of pulsed corticosteroid treatment. Focusing on these selected parameters, we did not observe significant changes associated with monthly IV-MP. However, this does not argue against a lasting immunological effect of pulsed steroids, which potentially could be measured by additional markers.

### Clinical prospects for pulsed glucocorticoids in MS

We are aware that the small number of patients studied and lack of long-term data prohibit direct clinical consequences from this study and we do not suggest pulsed corticosteroids as routine treatment. However, our study demonstrates an anti-inflammatory effect with almost no relevant short-term side effects, and no indication for adrenal insufficiency or osteopenia, in line with recently published data [43]. Our results thus support further research [17].

## Conclusion

In relapsing-remitting multiple sclerosis, once-monthly IV methylprednisolone reduces inflammatory activity as measured by the best currently available surrogate measure (high-frequency contrast enhanced brain MRI). The effect is sufficient to support further study of this approach in certain groups of patients.

## Competing interests

The author(s) declare that they have no competing interests.

## Authors' contributions

FTB, TK: Trial design, drafting of ethics submission; general organization; data collection and analysis; initial drafting of manuscript; DPA: Trial design, MRI data acquisition and analysis; participated in initial drafting of manuscript; FTB, TK, MS, CT: patient selection, information, enrollment, clinical follow-up, endocrinological testing, oversight of patient safety; ES: Assessment of MRI imaging quality, blinded MRI data analysis; MB, AW: Volumetric MRI analysis (T2-LL and BFV); AY: Trial design (Statistics), data analysis; UH, MD: Extraction, verification and statistical analysis of comparison group data (SLC database); FH: Assessment and interpretation of neuroendocrine testing; MU, FW: Laboratory analyses and interpretation of hormone, cytokine and adhesion molecule concentrations. All authors read and approved the final manuscript.

## Pre-publication history

The pre-publication history for this paper can be accessed here:



## Figures and Tables

**Table 1:** Demographic characteristics and primary outcome measure, the number of active lesions/scan (Mean [SEM])

**Patient**	**Age**	**Sex**	**EDSS**	**Duration of disease**	**Mean number of active lesions per monthly scan**		**Change, mean lesion number**
		F/M		years	baseline period	treatment period	%

All patients	32.7 [1.4]	8/1	1.5 [0.33]	6.3 [2.0]	**3.55 [0.78]**	**2.15 [0.69]**	- 39.4 p = 0.013*

01	25	F	0	1	1	0,5	- 50
02	32	F	0	2.5	1.83	0	- 100
03	31	F	2.0	5	7.67	5.83	- 34
04	34	F	1.5	4	2.33	2.83	+ 21
05	34	F	1.0	0.3	6	3.67	- 38
06	36	M	2.5	11.3	4.5	0.67	- 85
07	40	F	2.5	15	1	0.17	- 83
08	33	F	2.5	15	4.83	4.17	- 14
09	30	F	1.5	3	2.67	1.5	- 38
					**Median change**		**- 43.8 %**
					**Mean change**		**- 46.4 %**

* p for testing baseline vs. treatment period (Wilcoxon Matched-Pairs Signed-Ranks test for related samples).

**Table 2:** T2 lesion load and brain fractional volume (Mean [SEM]).

	**Baseline period**			**Treatment period**			
	Month 1	Month 6	Change (mth 6 vs. mth 1)	Month 7	Month 12	Change (mth 12 vs. mth 7)	Change (mth 12 vs. mth 6)

T2 lesion load (ml)	19.93 [4.8]	22.49 [4.64]	+ 13 % p < 0.001	20.68 [4.23]	17.70 [4.17]	- 14 % p = 0.001	- 21% p = 0.001
BFV (%)	86.4 [1.4]	86.1 [1.8]	- 0.35 % n.s.	85.9 [1.9]	83.9 [1.8]	-2.33 % n.s.	-2.56% n.s.

Multivariate Wilk's test for T2 lesion load, p = 0.002, and for BFV, p = 0.086; p values given in the table are for univariate F tests.

**Table 3:** Mean [SEM] of plasma hormone concentrations during the baseline and treatment period, and results of neuroendocrinological tests

	**Baseline period**	**Treatment period**	p*
**Plasma hormone concentrations (means of monthly tests)**			
Prolactin (ng/ml)	10.2 [0.8]*	8.7 [0.7]*	0.027
Cortisol (ng/ml)	114.4 [23.2]	122.4 [22.8]	n.s.
ACTH (pg/ml)	17.4 [2.3]	19.9 [4.3]	n.s.
**DEX-CRH test**			
CORT max (ng/ml)	39.7 [8]	41.1 [14]	n.s.
CORT AUC	147.5 [30]	152.8 [55]	n.s.
ACTH max (pg/ml)	20.4 [5]	22.5 [11]	n.s.
ACTH AUC	49.4 [7]	46.6 [11]	n.s.
**ACTH test**			
ACTH base (pg/ml)	24.40 [12.21]	28.69 [7.23]	n.s.
CORT base (ng/ml)	175.1 [22.6]	169.9 [22.4]	n.s.
CORT 60 min (ng/ml)	330.7 [59]	275.8 [26.5]	n.s.
Delta Cort (ng/ml)	149.70 [35.67]	163.00 [32.40]	n.s.

* p for comparisons of baseline vs. treatment periods, MANOVA

**Table 4:** Plasma cytokine receptor and VCAM-1 concentrations (Mean [SEM])

	**Baseline period**	**Treatment period**	p*
sTNF-R I (ng/ml)	1.4 [0.05]	1.4 [0.09]	n.s.
sTNF-R II (ng/ml)	3.3 [0.3]	3.4 [0.2]	n.s.
IL-1 ra (pg/ml)	262.9 [43.2]	246.4 [37.8]	n.s.
sVCAM-1 (ng/ml)	430.7 [28.9]	484.6 [52.6]	n.s.

* p for comparisons of baseline vs. treatment periods, MANOVA
